# Simulation Theory Applied to Direct Systematic Observation

**DOI:** 10.3389/fpsyg.2017.00905

**Published:** 2017-06-08

**Authors:** Rumen Manolov, José L. Losada

**Affiliations:** Department of Social Psychology and Quantitative Psychology, Faculty of Psychology, University of BarcelonaBarcelona, Spain

**Keywords:** direct observation, time sampling, alternating renewal process, prevalence, interval recording

## Abstract

Observational studies entail making several decisions before data collection, such as the observational design to use, the sampling of sessions within the observational period, the need for time sampling within the observation sessions, as well as the observation recording procedures to use. The focus of the present article is on observational recording procedures different from continuous recording (i.e., momentary time sampling, partial and whole interval recording). The main aim is to develop an online software application, constructed using R and the Shiny package, on the basis of simulations using the alternating renewal process (a model implemented in the *ARPobservation* package). The application offers graphical representations that can be useful to both university students constructing knowledge on Observational Methodology and to applied researchers planning to use discontinuous recording in their studies, because it helps identifying the conditions (e.g., interval length, average duration of the behavior of interest) in which the prevalence of the target behavior is expected to be estimated with less bias or no bias and with more efficiency. The estimation of frequency is another topic covered.

## Introduction

Observation as a means of gathering data has been and is still present across disciplines and contexts related to psychological processes, including clinical psychology (Langer et al., 2016), work-related behaviors (Beck et al., 2016), family interactions (Dishion et al., 2016), social competence in childhood (Vaughn et al., 2016), sports (Castañer et al., 2016), primatology (Dolado et al., 2016), and ethology in general (Pasquaretta et al., 2016). Observation is also the most frequently used means for gathering data in single-case designs in which the behavior of individuals usually takes place in free-operant contexts (Pustejovsky, 2015). In the present text, the focus is put on *direct* observation, which is considered direct in two senses (Fassnacht, 1982): there is nothing between observer and observed (e.g., no interview or questionnaire is used) and records are compiled immediately after the observation session. In that sense, Ayres and Gast (2010) distinguish direct observation from automated-quantitative recording (that does not require human observers) and direct measurement of permanent products (such as exams or reports elaborated by the participants).

In the following sections we present an example of an observational study, in the context of which we illustrate the decisions that need to be made when conducting such an investigation: (a) choose observational designs; (b) choose what to code; (c) decide whether time sampling is required; (d) choose an observational recording procedure. Afterward, we focus on the latter point; specifically, we describe the method used for performing the simulations for studying how well prevalence and frequency of the target behavior are estimated in different observational recording procedures. We comment on the way in which the results of the simulations are implemented into interactive graphs, how these graphs can be used and what their main results are.

### An Example

In an observational study, the aim is to focus on spontaneous behavior taking place in the natural environment of this target behavior and without modifications being introduced by the researcher. Specifically, the context of the example is Attention Deficit Hyperactivity Disorder (ADHD), due to its relatively high and maintained prevalence across countries and decades (Polanczyk et al., 2014). Moreover, the diagnostic criteria for ADHD are largely based on directly observable behaviors (American Psychiatric Association, 2013).

The aim of the study is to obtain initial information about a class of students, for whom the teacher claims that the number of interruptions and inappropriate behaviors is excessive, according to his perception. Specifically, the objective is defined as estimating the proportion of time in which the students are involved in off-tasks behaviors and on-task behaviors. Subsequent evaluation is planned for future research assessing whether the relative duration of off-tasks behaviors is excessive and whether they are systematically related to any of the students for which there is a suspicion by the teacher that they might present problems with deficit of attention or impulsivity.

### Decision #1: Choose an Observational Design

The design in Observational Methodology is the strategy determining the course of action or sequence of decisions about how to collect, organize and analyze the data, always subordinate to the objectives of the study (Anguera et al., 2001). The purpose of an observational design is to identify the axes of time (when to record: in a continuous or discontinuous way?), behaviors (what to record: one or several target behaviors?) and subjects (who to record: one or several participants?) involved in an investigation, in order to be able to propose the best strategy in an observation situation.

**FIGURE 1:**
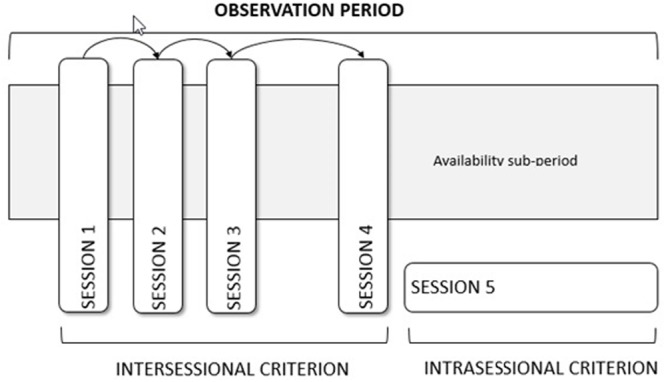
Representation of intersessional and intrasessional time sampling.

In the math class studied there are 10 students. According to the subject axis, a nomothetic design (Allport, 1942; Anguera et al., 2001) is used, given that all children are observed. According to the behavioral axis, a multidimensional design is used, given that there are several different specific behaviors coded as “off-task” or “on-task” (see the “Decision #2: Choose What to Code” subsection). According to the time axis (see **Figure 1**) and the inter-sessional criterion, the design is a “tracking” one (also referred as “follow-up” design), as several sequential sessions are to be recorded. According to the time axis and the intra-sessional criterion, time sampling has to be used, as discussed in the subsection entitled “Decision #3: Decide Whether Time Sampling Is Required.” The beginning and end of the observational sessions (i.e., the uninterrupted time of recording) are defined according to the duration of the math classes.

### Decision #2: Choose What to Code

Systematization of the recordings consists in expressing in observable terms all the information contained in behaviors or events, in order to improve objectivity. The behavioral units (i.e., the minimal behavioral manifestation that is considered meaningful) can be distinguished according to their duration, being either “states” (longer units, for which duration matters) or “events” (brief events, for which duration is not recorded; Altmann, 1974). Additionally, it is possible to distinguish the behavioral units according to their content, being “structural” (a physical movement or location, defined in time and space), “functional” (consequence of the structural units on the physical or social environment), or “causal” (causes of the structural units). Finally, the behavioral units can be classified according to their degree of abstraction, leading to “molecular” categories based on Weick’s (1968) response levels: verbal, vocal, gestures, and proxemics behavior or to “molar” categories (complex combinations of these response levels with a greater degree of abstraction, implying a certain amount of inference about the intentions).

For instance, Ardoin and Martens (2000) adapted Barkley’s (1990) Restricted Academic Situation and distinguished the following categories: off-task (interruption of the child’s attention from the task to engage in another behavior such as breaking eye contact with the worksheet), fidgeting (repetitive, purposeless movement of the legs, feet, arms, hands, fingers, buttocks, or trunk), vocalization (verbal noises), plays with objects (touching objects not directly related to the task, desk or child’s own body), and out of seat (child’s buttocks breaking contact with the seat). Similarly, yet slightly different, Stahr et al. (2006) mention as examples of “off-task behavior” the repetitive pencil tapping, head or leg shaking and fidgeting, drawing, gazing around class; leaving the assigned instructional area, and making audible vocalizations not related to the instructional task. Stahr et al. (2006) define “on-task behavior” as attending to or participating in instructional activities as requested by classroom staff (e.g., looking at the teacher while she was instructing, doing or attempting the assigned task, seeking assistance, and following directions). Therefore, the on-task and off-task behaviors refer to different response levels (i.e., they are “molar” categories), coded according to their relation to the academic task taking place at any given moment. Moreover, the focus is put on the function of the behavior rather than its location or the specific movement in any part of the body; thus, the units are “functional”. Finally, whereas some of the specific instances of on-task behavior can be “events” (e.g., shifting the gaze from the book to the blackboard), the “on-task behavior” category itself is rather a “state,” given that it is expected to have a certain duration.

### Decision #3: Decide whether Time Sampling is Required

In the running example, carrying out the observational study involving the direct presence of observers in the environment would require an authorization from the school. One approach would be “recording activated by transitions” (RAT), in which the observer is coding every transition from one category to another, optionally recoding duration times as well, without any time-related divisions of the observation session. However, a RAT would require video parents’ authorization for videotaping. Therefore, time sampling would be required. When the recording rule is conceptualized as “recording activated by units of time” (RAUT), the observation session is divided into many short intervals in which an observer determines if an event occurs (Barlow et al., 2009). These intervals are usually of constant duration, although in some cases intervals with variable duration are also possible (Test and Heward, 1984; Ayres and Gast, 2010). The main types of observational recording procedures that follow a RAUT rule are momentary time sampling (MTS, in which only the category taking place at the end of the time interval is recorded), partial interval recording (PIR, in which any category appearing at any point during the time interval is recorded) and whole interval recording (WIR, in which an occurrence is recorded only in case one category takes place throughout the whole interval) (Arrington, 1943; Hutt and Hutt, 1970; Cooper et al., 2007). In terms of taxonomies, Suen and Ary (1989) refer to PIR and WIR as “semi-continuous” recording and to MTS as “discrete” recording, whereas other authors (e.g., Rapp et al., 2011) refer to MTS, PIR, and WIR as “discontinuous” recording. The main features of the MTS, PIR, and WIR are described in **Table 1**.

**Table 1:** Main features of the observational recording procedures following a recording activated by units of time (RAUT) rule.

**Feature**	**Momentary time sampling**	**Partial interval sampling**	**Whole interval sampling**
Recording rule	Code the category that occurs at the end of the interval	Code any category that occurs during any moment within the interval	Code the category that occurs during the whole interval or as per Hutt and Hutt (1970) code the predominant category
Need for observer attention during the whole interval	No, only at the end of the interval	Yes, unless all categories in the coding scheme already took place at least once	Yes, unless the category present since the beginning of the interval stops occurring before its end
Minimum number of categories that can be coded in an interval	1	1	0
Maximum number of categories that can be coded in an interval	1	As many as categories present in the coding scheme	1
Coding of several occurrences within a single interval	Coded as one occurrence, only if taking place at the end of the interval; otherwise, 0	Coded as one occurrence.	Coded as zero occurrences, assuming that a non-occurrence takes place in between
Coding of a single occurrence spreading over two intervals	Coded as one occurrence, assuming that it takes place at the end of the interval; coded as two occurrences if it last until the end of the second interval	Coded as two occurrences	Coded as zero occurrences, unless it takes place during the whole interval (coded as 1 or 2).

Opting for MTS, PIR, or WIR as feasible alternatives to continuous recording is justified on the basis that all these recording procedures have been commonly used in a variety of disciplines (e.g., Mudford et al., 2009, report that discontinuous recording was used in 45% of the articles reviewed; Adamson and Wachsmuth, 2014, report that MTS was used in 9% of the articles using direct observation and a time-based system like PIR or WIR was used in 48% of the studies, versus 39% using an event-based code). Moreover, MTS, PIR, and WIR may inform about whether a behavior is likely to occur at the beginning, mid, or end of an observation period, which cannot be assessed via event coding only.

**FIGURE 2:**
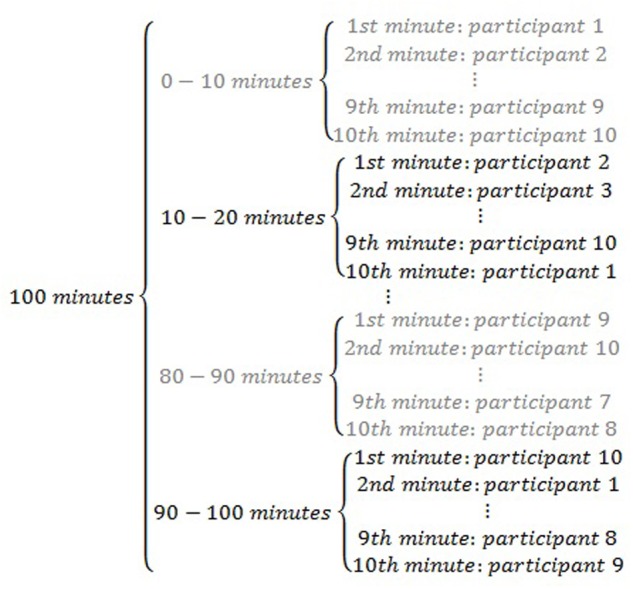
Graphical representation of within-session sampling of participants to be the focus of observation: alternating multifocal sweep.

Given that there are several participants to be observed, this can be achieved using multifocal sweep sampling, and more specifically, its alternating variant. This within-session sampling of focal participants takes places as follows. The observational session lasting for 100 min is divided into ten 10-min fractions. In the first fraction, during the 1st minute participant 1 is observed, during the 2nd minute participant 2 is observed, and so forth up to participant 10 being observed during the 10th minute. In the second fraction, during the 1st minute participant 2 is observed, during the 2nd minute participant 3 is observed, and so forth up to participant 10 being observed during the 9th minute and participant 10 being observed in the 10th minute. The sequence of observing the participants continues accordingly up to the 10th fraction in which during the 1st minute participant 10 is observed, during the 2nd minute participant 1 is observed, and so forth up to participant 9 being observed during the 10th minute. This alternating multifocal sweep sampling (represented on **Figure 2**) ensures that all individuals are observed in all fractions and, additionally, that all individuals are observed in different parts of the fractions (i.e., not always at the beginning or at the end). Subsequently, it is necessary to choose the interval length and the specific observational recording procedure to use (see next section).

### Decision #4: Choosing A Raut Observational Recording Procedure

There are three reasons why the choice of a discontinuous recording procedure is important. First, they are expected (and have been shown) to present random or systematic errors, due to the fact that these procedures do not record the frequency and duration of each category (Gardenier et al., 2004). Second, it has been shown (Rapp et al., 2011) that the type of observation recording procedure used on the same real behavioral stream is related to the degree of interobserver agreement (IOA). This finding suggests that high values of IOA are not necessarily the result of high concordance between data collected by two independent observers, but could also stem from procedural features. Third, inaccuracy of MTS, PIR, and WIR in estimating count and duration also has an effect on subsequent analyses performed for giving an answer to the research question of interest (e.g., see Ledford et al., 2015, for results related to estimating effects in single-case designs; Barlow et al., 2009). Accordingly, there have been efforts to propose effect size indices, whose values do not depend on the observation recording procedure (Pustejovsky, 2015).

The factors that have been related to the presence of error are: (a) the type of time sampling method used (Powell et al., 1977; Simpson and Simpson, 1977; Murphy and Goodall, 1980; Green et al., 1982; Gardenier et al., 2004; Alvero et al., 2007; Rapp et al., 2008; Devine et al., 2011); (b) the length of the intervals used (Dunbar, 1976; Leger, 1977; Powell et al., 1977; Mansell, 1985; Mudford et al., 1990; Alvero et al., 2007, 2011; Rapp et al., 2008; Devine et al., 2011) and (c) factors related to the categories of interest, such as its frequency (McDowell, 1973; Powell et al., 1977; Murphy and Goodall, 1980; Green et al., 1982; Gardenier et al., 2004; Alvero et al., 2011) and duration (Murphy and Goodall, 1980; Sanson-Fisher et al., 1980; Green et al., 1982; Ary and Suen, 1986). In general, it has been observed that when the duration of the interval (τ) is small relative to the duration of the category and the spaces between categories, the estimates will be more precise (Suen and Ary, 1989). The number of factors and the number references provided suggests that choosing a discontinuous recording procedure and, additionally, choosing an interval length are not necessarily straightforward tasks. The interactive graphs we created and implemented in a web page are intended to provide guidance for this specific decision in the process of conducting an observational study.

### Aim of the Article

Given that observation is commonly present in research and it is also included in the curricula of university majors such as Psychology and Educational Sciences, it is important to illustrate the conditions (e.g., interval length, average duration and prevalence of the behavior of interest) in which MTS, PIR, and WIR are expected to perform well when estimating estimate the frequency and prevalence of the behavior of interest. Specifically, we here describe the development of interactive graphs available in a free web page, with the aim to make accessible to students and applied researchers the complex simulation evidence, taking into consideration several factors at a time.

## Method

### Justification of the Need for Simulation

Simulations offer several advantages over the analysis of observational records obtained from real situations. First, simulations entail knowing the truth about the parameters of the underlying process from which the observed behavioral streams arise. More concretely, the researcher can specify the average duration of the behavior each time that it occurs (i.e., how long or how short are the individual occurrences, on average) or the proportion of time that it takes place (i.e., what is the prevalence of the behavior). Second, an evaluation of the sampling methods with a simulation eliminates the error attributable to the human observer. The possible error produced in the records by the observer can be indirectly attributed to a series of variables such as biological factors, psycho-social factors, reaction time, motivation, behavior perceptibility (Repp et al., 1976; Tyler, 1979; Green et al., 1982; Saudargas and Zanolli, 1990; Murphy and Harrop, 1994; Taylor et al., 2012). Third, the measurement error can be quantified either in terms of absolute error values (i.e., difference between estimated and actual durations) or in terms of relative error values (i.e., the difference expressed as a proportion of the actual durations of the events; this is the option we followed here).

### Data Generation Model

For generating the behavioral stream of occurrences and their duration we used the alternating renewal process (ARP) model (Pustejovsky and Runyon, 2014), implemented in the *ARPobservation* package for R (R Core Team, 2016). ARP treats both the length of behavioral events and the interim times (i.e., interresponse time between events) as random quantities (Pustejovsky and Swan, 2015).

The review of simulation studies performed by Pustejovsky and Runyon (2014) showed that most studies followed a procedure that agrees with the ARP model, whereas others mostly followed a *random onset model* in which the point of onset for a behavioral event is chosen at random repeatedly, on the basis of a predetermined duration per occurrence, and usually avoiding overlaps (e.g., Ledford et al., 2015). Another procedure followed in previous research (Rapp et al., 2011) is to use real data gathered via continuous recording and then to convert this data to interval measures on the basis of MTS, PIR, or WIR.

The main advantage of the ARP model and the *ARPobservation* package is that it mimics the actual process in which there is first a behavioral stream and then data are gathered according to a predetermined procedure (continuous recording, MTS, PIR, or WIR). Moreover, the ARP model offers great flexibility in simulating behavioral streams with different characteristics (Pustejovsky and Runyon, 2014).

The assumptions of the ARP model include (Pustejovsky and Swan, 2015): the event duration times corresponding to the same observation session are assumed to be identically distributed; the interim times corresponding to the same observation session are assumed to be identically distributed^1^; the length of the next event or interim time does not depend on the sequence of events leading up to it; there is a constant probability that an event is occurring at any given point in time during the observation session (i.e., the behavior stream is in equilibrium).

### Data Generation Parameters

The following are the relevant simulation parameters that describe the main characteristics of the observational situation:

(a) Session duration: set to 10, 20, 30, and 60 min. Previous studies included sessions of 10 min (Ledford et al., 2015), or 10, 15, and 30 min (Rapp et al., 2011). According to the review performed by Pustejovsky and Runyon (2014) the range of session durations is between 10 and 300 min, with most common lengths being 30 or 60 min.
(b) The prevalence of the behavior of interest (π) is defined as proportion of duration with respect to whole observation session length. We used the range from 5 to 95% in steps of 5%. Ledford et al. (2015) varied prevalence from 10 to 70%, Pustejovsky and Runyon (2014) provided an illustration with values from 1 to 99%, in steps of 1%, and the prevalence from Rapp et al.’s (2011) real data sets ranged from 10 to 93%.
(c) The incidence per time unit is defined as the average number of times that a behavior occurs, for instance, per minute. Pustejovsky and Runyon (2014) provided an illustration with values ranging from 0.1 to 0.5. Incidence is not manipulated directly in the *ARPobservation* package; we rather tallied the occurrences and divided the sum by the observation session length, measured in minutes. Pustejovsky and Runyon (2014) define incidence, within the ARP framework, as being equal to 1/(μ+λ), where μ is the average event duration and is the average interim time. In our simulations, incidence ranged from 0.1 to 3.2 (according to the prevalence of the behavior) for μ = 18 s and from 1.5 to 28.6 (according to the prevalence of the behavior) for μ = 2 s.
(d) Average event duration (μ), also referred to as mean bout duration or average “duration per occurrence” (DPO): ranging from 2 to 120 s in our simulation. Comparatively, Ledford et al. (2015) set DPO to 2 or 10 s, whereas Pustejovsky and Swan (2015) provide an illustration with DPO = 6 s. In the review performed by Pustejovsky and Runyon (2014), the most common DPOs were in the range of 1 to 120 s, with three of the 14 studies using greater values of the maximum DPOs, up to 500 s.
(e) Average interim time (λ): this parameter was determined according to the previously defined average DPO and prevalence. Specifically, given that Pustejovsky and Runyon (2014) define prevalence, within the ARP framework, as being equal to π = μ/(μ+λ), then λ = (μ-μπ)/π. Thus, for instance, for π = 0.5, the average interim time was equal toμ, whereas for π = 0.3 the average interim time ranged from 4.67 s (for μ = 2 s) to 42 s (for μ = 18 s). Ledford et al. (2015) did not set interim times explicitly, as they apparently followed the random onset model rather than the ARP model. Pustejovsky and Swan (2015) use 12 s, with the most common values ranging from 2 to 60 s according to the Pustejovsky and Runyon (2014) review.
(f) For discontinuous recording, the interval length (τ) has to be set. We used interval lengths ranging from 2 to 20 s. Ledford et al. (2015) used intervals of 2 and 20 s as well, whereas Rapp et al. (2011) used 10 s, and Pustejovsky and Swan (2015) 5 and 20 s. A relevant aspect highlighted by Ledford et al. (2015) and Pustejovsky and Swan (2015) is whether the interval is longer or shorter than the average DPO, which is related to the degree to which estimates of count and duration obtained from discontinuous recording misrepresent the measures from continuous recording. Specifically, Ledford et al. (2015) studied interval size relative to DPO ranging from 0.33 to 3.33. In the interactive graphs we developed we also included a calculation of interval size relative to DPO for the specific combination of conditions selected by the user. For the shortest interval (τ = 2 s) and longest behavior (μ = 18 s), the ratio τ/μ is 0.11, whereas for the longest interval (τ = 20 s) and shortest behavior (μ = 2 s), the ratio τ/μ is 10.

**FIGURE 3:**
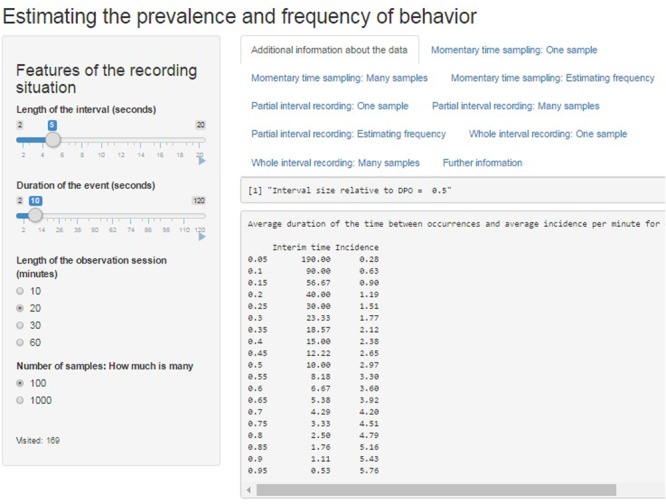
Screenshot of the web application created using Shiny. The average interim time and the average incidence per minute can be seen for each value of prevalence, considering the average duration of the event.

**Figure 3** illustrates how the parameters can be selected in the web application and it also shows how the website presents the information about the ratio τ/μ, and about average interim time and incidence per minute for each of the values of prevalence.

### Data Analysis

With the ARP model it is possible to assess the performance of discontinuous recording in two different ways (Pustejovsky and Runyon, 2014). On the one hand, it is possible to compare the measures from discontinuous recording to the ones that would be obtained in continuous recording. In this case, we would be assessing how well the observed behavior is represented, taking into account that MTS, PIR, and WIR entail time sampling within the observation session. This approach takes into consideration the fact that continuous recording does not contain intrasession sampling error (Suen and Ary, 1989). On the other hand, it is possible to compare both the measures from discontinuous recording and the measures from continuous recording to the parameters that generate the behavior stream. According to this latter approach, the behavior observed in a given session and measured via continuous recording is only a realization of the underlying process, as selecting the moments for the observation sessions also involves time sampling of the behavior of the organism studied. This approach takes into consideration the fact that continuous recording may contain intersession sampling error (Suen and Ary, 1989). Both kinds of comparison are possible with the interactive graphs created.

The interactive graphs offer results for 1, 100, or 1000 samples. The results for 1 sample illustrate what could happen in any given study (in which the results from continuous recording need not match perfectly well the underlying process generating the behavior in a given observation session), whereas the results for 100 and especially for 1000 samples are more informative of the general performance of the discontinuous recording techniques as compared to continuous recording. When the results for 100 or 1000 samples are represented graphically, apart from the average value, we also provide information about the scatter: one or two standard deviations away from the mean, represented in orange and red, respectively.

The following terms are relevant for the results illustrated in the interactive graphs:

(a) Pseudofrequency: PF = r + n_01_, where *r* is equal to 0 when the first interval in the observation session is not marked and it is equal to 1 when this interval is marked as denoting occurrence of the behavior, and n_01_ is the number of transitions from non-occurrence (unmarked interval) to occurrence (marked interval). According to Suen and Ary (1989), *PF* would be an unbiased estimator of frequency when the interval is shorter than the shortest behavior duration and shorter than one half the shortest interim time.
(b) Modified frequency (θ) = number of intervals for which the occurrence of the behavior of interest is marked when using MTS, PIR, or WIR. The modified frequency would be the most straightforward way of estimating frequency, although evidence has shown that it is imperfect. We have included this way of estimating frequency for MTS and PIR in order to enable exploring whether it is appropriate in any of the conditions tested. Moreover, the modified frequency is also in the basis of estimating prevalence; in general, it is assumed that prevalence is estimated as 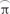
= θ/n, where *n* is the number of intervals into which the observation session is divided. However, for PIR and WIR corrections have been proposed (Suen and Ary, 1989): 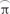
_PIR_ = (θ - PF)/n and 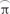
_WIR_ = (θ + PF)/n, respectively.
(c) For PIR we also applied a formula for estimating frequency that is not based solely on the modified frequency (Altmann and Wagner, 1970):
  $$\begin{array}{c} \overset{⌢}{f}=-\left(n\times \ln\left(1-\frac{\theta }{n}\right)\right) \end{array}$$
  This formula is expected to function well when: (a) the behavior of interest is an event (i.e., it has a very short duration, practically equal to zero), and (b) the probability of occurrence of the behavior of interest is independent of the time that has passed since the last time it occurred, as the case for a Poisson distribution. In relation to point (b), in the ARP model “[a]ll interim times and all event durations are generated in a mutually independent manner, which means that the length of a given event is influenced neither by the length of previous events nor by how long it has been since the last event ended” (Pustejovsky and Runyon, 2014, p. 213).

Finally, the amount of error when estimating prevalence is quantified as relative bias, using the formula: (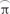-π)/π, where π is the value of the simulation parameter for prevalence and 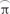 is the estimated obtained using MTS, PIR, or WIR. For PIR and WIR, relative bias is computed separately for estimating prevalence as 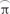
= θ/n or as 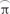
_PIR_ = (θ - PF)/n and
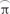
_WIR_ = (θ + PF)/n.

### Development of the Application

The illustrations are based on the ARP model and the *ARPobservation* package and have been prepared using Shiny applications^2^, for two reasons. First, from the perspective of the interested reader, Shiny is freely available and user-friendly, given that the only actions required to obtain the graphical and numerical results are selecting options from the left-hand side menus and clicking the tabs in the upper part of the browser (see **Figure 3**). Second, from the perspective of the researcher and developer, Shiny communicates easily with R^3^, which is the free platform in which the ARP model is implemented. This communication is made efficient thanks to RStudio^4^. The interactive graphs and tables are available at http://jlosada.shinyapps.io/Prevalence.

## Output of the Application

### Obtaining the Results

When accessing http://jlosada.shinyapps.io/Prevalence the user can manipulate the options at the left of the web browser in order to specify several features defining the observation session: (a) length of the observation session; (b) length of the interval in seconds; (c) the average duration of the behavior of interest in seconds; and (d) the number of samples when presenting the results of more than one sample. When a selection is made (or with the default selection), information is provided in the initially active tab called “Additional information about the data.” In the first row, the ratio of the interval length (τ) to average DPO (μ) is provided. Afterward, a table is presented containing the average interim time (λ) and the average incidence per minute for each of the values of prevalence (π) of the behavior of interest. A screenshot including this information is provided in **Figure 3**.

**FIGURE 4:**
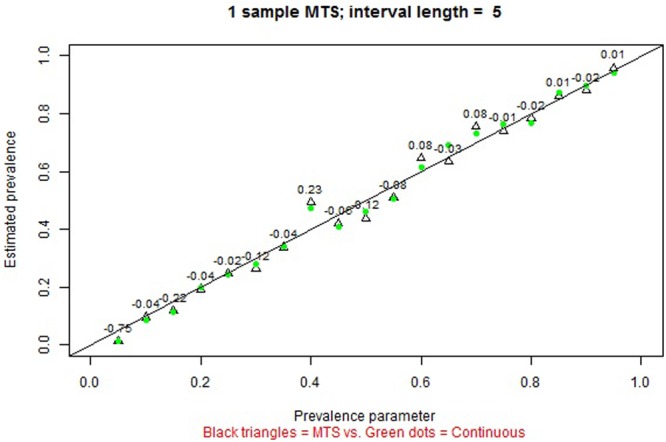
Screenshot of the web application created using Shiny. Prevalence of a behavior with average duration per occurrence of 8 s as estimated in a single observation session of 20 min, using continuous recording (green dots) and momentary time sampling [MTS] (empty triangles) based on a 5-s interval. The numerical values represent the relative bias of the estimation using MTS.

**FIGURE 5:**
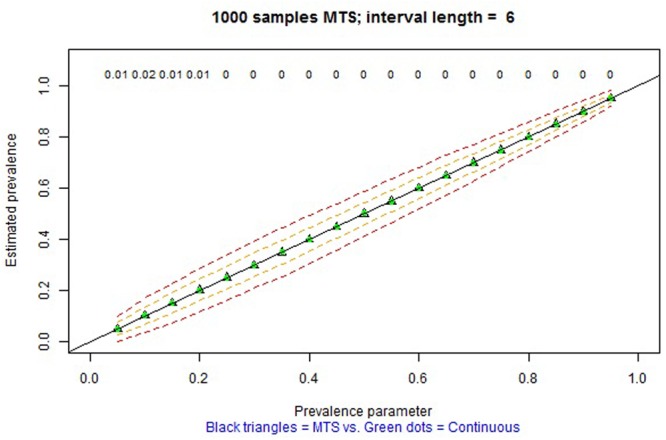
Screenshot of the web application created using Shiny. Average prevalence of a behavior with average duration per occurrence of 8 s as estimated in 1000 observation sessions of 20 min, using continuous recording (green dots) and MTS (empty triangles) based on a 6-s interval. The dashed lines represent one and two standard deviations above and below the average estimate by MTS. The numerical values represent the relative bias of the estimation using MTS.

**FIGURE 6:**
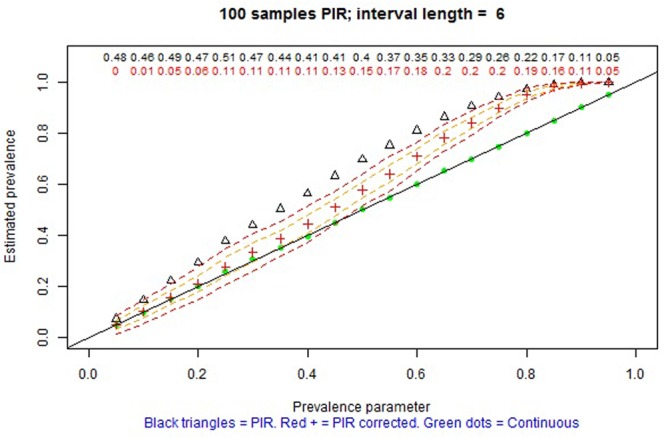
Screenshot of the web application created using Shiny. Average prevalence of a behavior with average duration per occurrence of 12 s as estimated in 100 observation sessions of 60 min, using continuous recording (green dots) and partial interval recording (PIR) (empty triangles without the correction; red crosses with the correction) based on a 6-s interval. The dashed lines represent one and two standard deviations above and below the average of the corrected estimates by PIR. The numerical values represent the relative bias of the estimation using PIR: black values refer to using the modified frequency in the numerator, whereas red values refer to using modified frequency minus pseudofrequency in the numerator.

**FIGURE 7:**
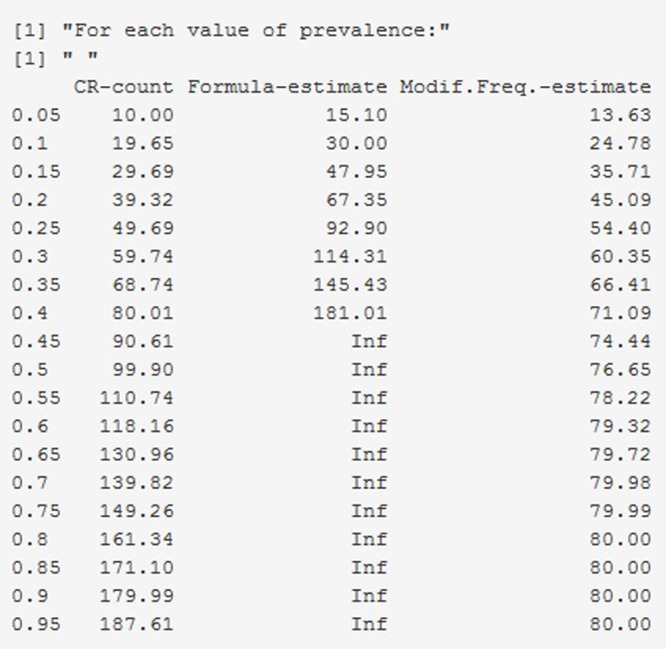
Screenshot of the web application created using Shiny. Frequency estimates of a behavior with average duration per occurrence of 6 s as estimated in 100 observation sessions of 20 min, using PIR) based on a 15-s interval (i.e., the rate of interval length to duration per occurrence is 2.5).

The remaining tabs offer two types of information. On the one hand, there are graphical representations of the estimated prevalence (on the ordinate) for each simulation parameter π on the abscissa (e.g., **Figures 4, 5** for MTS and **Figure 6** for PIR). On the other hand, there are tabular representations of the estimated frequency (third column for MTS; third and fourth columns for PIR) compared to the average frequency as determined by continuous recording (second column), for each value of prevalence (e.g., **Figure 7** for PIR). The information is obtained by clicking on the tabs, with several seconds required for the corresponding simulations to take place and to provide the graphical or tabular output.

### Using the Application for Pedagogic Purposes

An initial pedagogic purpose could be to illustrate the concept of sampling variability, clicking on any of the three tabs illustrating the results of one sample. When comparing the results of the recordings in a single observation session with the simulation parameters that defined the underlying process generating the behavioral stream, the graphs make obvious that not even continuous recording is absolutely perfect for estimating prevalence. This is due to the fact the behavior observed in a given session is only a sample. The results for MTS and continuous recording are usually similar for short intervals and when the average DPO is longer than the interval used in MTS. **Figure 4** presents an example.

A second purpose could be to illustrate the degree to which there is overestimation or underestimation of prevalence, according to the interval length (τ) and average DPO (μ), while also considering the actual simulation parameter π. For that purpose, the play buttons for τ and μ can be used in order to provide a visual impression of the importance of these factors and how they interact. The play buttons are useful when presenting the results for one sample, because the use of many iterations requires time and the play buttons are not practical anymore. However, the graphical representations generated on the basis of 100 or 1000 iterations can be saved and compared afterward by putting them side by side.

In general, over many iterations, when the comparison is performed with the simulation parameters that defined the underlying process generating the behavioral stream, prevalence is estimated without bias when continuous recording and MTS are used. For MTS, more precise estimates of prevalence (i.e., narrower standard deviation bands, as represented on the interactive graphs) are obtained for: (a) shorter intervals (i.e., smaller τ), (b) behaviors with shorter duration μ, and (c) longer observation sessions. **Figure 5** presents an example.

For PIR prevalence is overestimated. However, when the correction proposed by Suen and Ary (1989) is applied, this overestimation is attenuated, although not removed, consistent with the findings of Rogosa and Ghandour (1991). Complementarily, for WIR prevalence is underestimated, but the correction leads to attenuating this overestimation. For both PIR and WIR, in terms of bias, the averages of estimates are closer to the simulation parameters for: (a) lower actual levels of prevalence (π ≤ 0.3) than for higher ones, (b) shorter intervals in general (e.g., for τ = 2 s PIR provides practically unbiased estimates of prevalence), (c) smaller τ/μ ratio, as reported by Ledford et al. (2015), and (d) longer observation sessions. More precise estimates of prevalence are obtained for actual prevalence close to 0 or 1, due to the bounds of the index, and also for the three previously mentioned situations. **Figure 6** shows an example for one of the favorable conditions for PIR, but for which the estimation of prevalence is also biased.

For PIR, regarding the estimation of frequency via the formula by Altmann and Wagner (1970), the results obtained indicate that in no condition (not even when μ = 2 s) did the formula provide a good estimate of frequency, as computed via continuous recording. Actually, the results illustrated in the graphs are worse than the ones reported by Ledford et al. (2015), who used θ as an estimate of count and found that smaller counts were estimated better in longer intervals and larger counts were estimated better in shorter intervals. In few situations meeting these conditions the estimates of frequency using θ were within 10% of the actual count. **Figure 7** shows a snapshot of the table generated in the website, illustrating the abovementioned findings about these two ways of estimating frequency when using PIR.

### Using the Application for Applied Research Purposes

When the aim of the use of the Shiny application is to choose an appropriate interval for a given RAUT, there are several possible scenarios. First, if absolutely no prior information is available, the applied researcher would have to follow an approach similar to the one describe for the pedagogic use of the Shiny application.

**FIGURE 8:**
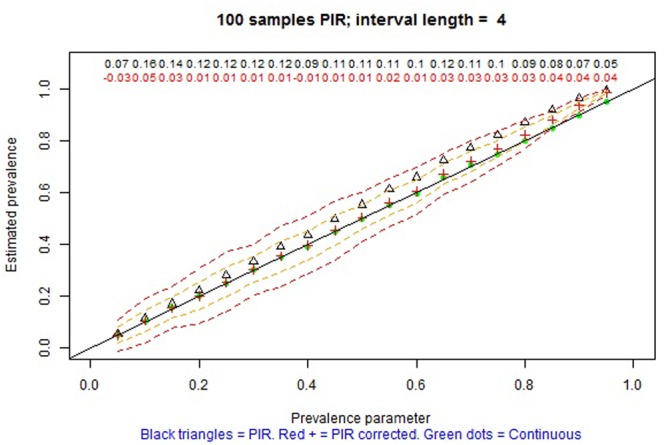
Screenshot of the web application created using Shiny. Average prevalence of a behavior with average duration per occurrence of 35 s as estimated in 100 observation sessions of 60 min, using continuous recording (green dots) and PIR (empty triangles without the correction; red crosses with the correction) based on a 4-s interval. The dashed lines represent one and two standard deviations above and below the average of the corrected estimates by PIR. The numerical values represent the relative bias of the estimation using PIR: black values refer to using the modified frequency in the numerator, whereas red values refer to using modified frequency minus pseudofrequency in the numerator.

Second, in some cases it is possible to have an empirically based expectation on the approximate prevalence of the behavior of interest. For instance, the estimated prevalence of on-task behavior for children with ADHD has been reported to be between 0.30 and 0.50 according to the moment of the observation session (Rapport et al., 2009), an average of 0.64 with a standard deviation of 0.06 (Junod et al., 2006), or as high as an average of 0.71 average with a standard deviation of 0.16 (Mahar et al., 2006). For such high values of expected prevalence, the even the 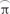
_PIR_ = (θ - PF)/n estimates of prevalence are always positively biased (e.g., see **Figure 6** and, specifically, the red crosses, denoting the estimates of prevalence, above the diagonal black line representing unbiased estimation, for prevalences greater than 0.2), but the overestimation is attenuated when the average DPO is μ ≥ 30 and τ ≤ 5 (e.g., see **Figure 8** and, specifically, the red crosses on the diagonal black line for practically all values of prevalence). If the there is no evidence for assuming μ ≥ 30, on the one hand, and τ ≤ 5 is judged not to be practical, on the other hand, then PIR should not be considered as an adequate observation recording procedure. In such a case, it would be advisable to use MTS instead of PIR.

**FIGURE 9:**
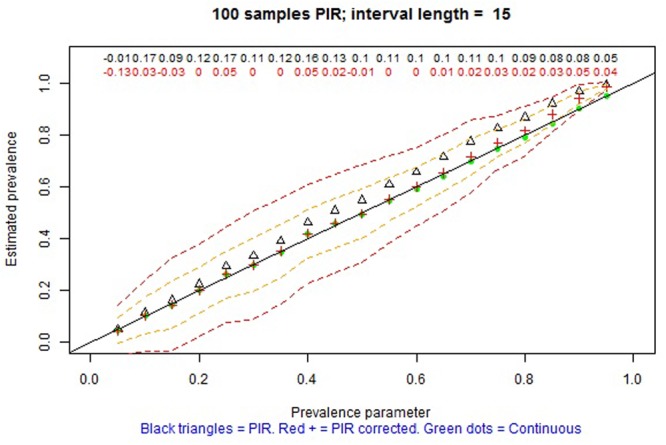
Screenshot of the web application created using Shiny. Average prevalence of a behavior with average duration per occurrence of 120 s as estimated in 100 observation sessions of 60 min, using continuous recording (green dots) and PIR (empty triangles without the correction; red crosses with the correction) based on a 15-s interval. The dashed lines represent one and two standard deviations above and below the average of the corrected estimates by PIR. The numerical values represent the relative bias of the estimation using PIR: black values refer to using the modified frequency in the numerator, whereas red values refer to using modified frequency minus pseudofrequency in the numerator.

A third situation would entail having information about both the likely range of prevalence and the average DPO, although the latter has been claimed to be seldom reported (Ledford et al., 2015). If we use the information from Rapport et al. (2009) that the average duration of on task behavior for children diagnosed with ADHD and low attention is 2 min (120 s), an interval length of τ = 15 (as actually used by Rapport et al., 2009) would be justified, as illustrated from **Figure 9** in which the estimates of prevalence (red crosses) are practically unbiased (i.e., close to the diagonal line).

### Summary of the Results of the Application

Concerning the estimation of prevalence and frequency, the evidence of the performance of discontinuous recording procedures is very complex, due to the fact that this performance is affected by many interacting factors. This complexity makes difficult summarizing the results via a simple rule. For instance, Ayres and Gast (2010) suggest that WIR is more appropriate when the behavior of interest is of low frequency and long duration, whereas PIR is appropriate for behaviors of high frequency and short duration, given that the frequency of long duration behaviors may be overestimated. This statement can be verified from the interactive graphs. Moreover, more nuanced knowledge can be obtained, as it can be verified that the frequency of short duration behaviors is also overestimated, for certain combinations of interval length τ and average behavior duration μ with τ > μ, when the prevalence π is relatively low (below 0.45 for some combinations of τ and μ or below 0.75 for other combinations). Regarding MTS, Ayres and Gast (2010) state that it is appropriate for behaviors with high frequency and long durations and that this recording procedure has a tendency to underestimate frequency and overestimate duration. Using the interactive graphs it can be shown that prevalence is actually not overestimated, whereas the underestimation of frequency is only present when the length of the interval is greater than the average duration of the event (τ > μ); in contrast, frequency is overestimated when the length of the intervals is shorter than the average duration of the event (τ < μ) and the estimation is unbiased when behavior and interval are of the same length.

Although the aim of the interactive graphs was to provide nuanced information, taking into account the specific interval lengths, DPOs, and prevalences, it should be noted that **Table 2** includes a necessarily simplified summary of the performance of the time sampling methods for estimating prevalence and frequency. This summary suggest that MTS can be recommended to be used when the aim is to estimate prevalence (e.g., **Figure 5**), especially when interval is short and when the average DPO of the behavior is short. These results concurs with previous findings regarding the lack of systematic bias (Tyler, 1979; Harrop and Daniels, 1986); specifically, Rogosa and Ghandour (1991) note that MTS is useful for estimating prevalence, but not incidence or event duration.

**Table 2:** Performance of the observational recording procedures following a recording activated by units of time (RAUT) rule.

Feature	Momentary time sampling	Partial interval sampling	Whole interval sampling
General summary of performance for estimating prevalence	Unbiased estimation; more efficient for shμ and when π is small	Underestimation, even with correction, but less severe when τ < μ and when π is small
General summary of performance for estimating frequency	Estimation via modified frequency: (a) underestimation when τ > μ; (b) overestimation when τ < μ; (c) unbiased estimation when τ = μ	Estimation via modified frequency: overestimation, unless τ > μ, but depending on π. Estimation via the formula by Altmann and Wagner (1970): more severe overestimation.	Not included in the application, as the literature review does not provide support.

*τ, interval length; μ, average duration per occurrence; π, prevalence.*

In contrast, the results concur with previous findings about PIR overestimating of the frequency and prevalence of the categories (Tyler, 1979; Harrop and Daniels, 1986), which is why Rogosa and Ghandour (1991) state that PIR does not provide useful information on incidence, prevalence, or event duration. More specifically, the results from the interactive graphs suggest that PIR can only be used for estimating prevalence in case 2 τ < μ and for π ≤ 0.3 (e.g., **Figure 6**). For WIR, the requirement is even more stringent: 3 τ < μ. This result is consistent with previous findings about the underestimation when using WIR being greater for longer intervals (Alvero et al., 2007). Thus, if the prevalence is not known beforehand and if the bout durations are relatively short, PIR and WIR should not be used when the objective is to estimate prevalence.

In terms of estimating frequency, this can be done without systematic error only when the average DPO is known and it is used for defining the interval length when using MTS. For PIR the requirements involve prevalence as well, which means that it is a less practical option. In summary, the choice of a time sampling method is an important one in order to avoid inaccurate descriptions of the degree to which the phenomena of interest are present or inaccurate comparisons, especially if different observational recording procedures are used for the different behaviors observed. For instance, Abikoff et al. (2002, p. 353) use MTS and WIR to obtain “behavioral rates” of children with ADHD and Junod et al. (2006) use MTS and PIR to estimate prevalence of several behaviors children with and without ADHD; in neither of the two cases is there any mention of average DPO or prevalence.

## Discussion

### Advantages and Limitations of the Application

The application constructed has several advantages. First, it is available online free of charge. Second, the application is user-friendly in the sense that no programming skills are required and the selection of the values of the factors defining the observational situation is made by clicking. Third, according to the review performed by Pustejovsky and Runyon (2014), the ARP model used for the simulation is a framework representing most of the simulation studies on observational data. Fourth, for obtaining the results of the simulation, it is not necessary to specify potentially unavailable information, such as the average incidence per minute. Accordingly, it is not strictly speaking necessary to know the average DPO beforehand, given that the user can select several likely values using the slider in the application. For the same purpose (i.e., not requiring specific knowledge about the expected prevalence), the graphical representations cover practically the whole range of possible prevalences. In that sense, it is not required to have information about the specific values of incidence, average DPO or prevalence to get a general insight of the interval lengths that are justified to be used. Fifth, the variety of parameter values for defining the observation situation (i.e., observation session length, average DPO, prevalence of the behavior of interest, interval length and the average interim time, incidence, and ratio of interval length to average DPO) is greater than the one present in recent simulation studies.

Besides strengths, it is especially important to dedicate space to the limitations of the application, taking into account the use of simulation as a basis (e.g., von Oertzen and Brandmaier, 2013). Regarding the limitations of the application, an initial technical limitation refers to the fact that the simulations are performed when the user selects the values defining the observational situations rather than accessing information (e.g., stored in data matrices) of already performed simulations. Therefore, it is not possible to always obtain instantly the results when performing 100 or 1000 iterations. Our calculations suggest that for 1000 iterations for MTS approximately 5 s are needed, whereas for 100 iterations for PIR require between 10 and 15 s. Second, we can mention as limitations the assumptions of the ARP model mentioned previously (i.e., the event duration times corresponding to the same observation session are assumed to be identically distributed and there is a constant probability that an event is occurring at any given point in time during the observation session) and to the fact that we used only one distribution (the exponential) for modeling event durations and interim times. Third, a limitation of the evidence provided in the Shiny application is related to the way in which the behavior stream is converted into strings of categories. Specifically, human error is not included in the simulation process and this represents a relevant future endeavor for modifying the *ARPobservation* package that is used as a basis of the simulations. Fourth, the graphical representations do not cover all possible combinations of average DPO and interval length. Therefore, as is the case for any simulation, the evidence cannot be considered as representing perfectly all real situations, but it can be used as an indication in absence of better simulation models or in absence of specific knowledge about interval lengths that have been proven to be useful for estimating the prevalence of given behaviors.

### Implications for Teachers and Methodologists

In order to improve the way in which knowledge is transmitted or, more accurately, the way in which students construct knowledge (Driver et al., 1994), there are already efforts focused on statistical topics, including specialized journals such as *Understanding Statistics.* However, some topics specific to observational methodology need more attention. In that sense, from the perspective of the teacher or methodologist, the three types of competence (McLagan, 1997; Kaslow, 2004) are involved in constructing and using the interactive graphs presented in the current text: (a) the fundament is the *attitude* to try to improve teaching methods; (b) specific *knowledge* is constructed by the teacher or methodologist in relation to the conditions (e.g., average duration per occurrence of the behavior, interval length, and ratio of the two) in which each of the discontinuous observation recording procedures perform best; and (c) methodological *skills* are developed by learning to use software specifically designed for simulating behavior in observation sessions and for using different recording procedures. Interactive graphs such as the ones presented here make possible a presentation of empirical findings that is both more detailed (i.e., covering a greater range of conditions) and more accurate (i.e., avoiding oversimplifications and representing the amount of bias present in the different conditions).

### Implications for Students and Applied Researchers

The same three types of competence are also involved from the perspective of the student or applied researcher: (a) the fundament is the *attitude* or disposition to follow the best possible practices when choosing the recording procedure to use for observing overt behaviors; (b) *knowledge* or subject matter is constructed, in this case, on the topic referring to the strengths and limitations of different observation recording procedures (continuous recording, MTS, PIR, and WIR); and (c) methodological *skills* or abilities are expected to be developed by getting acquainted with the simulation procedure followed for studying the quality of the measures obtained in MTS, PIR, and WIR (i.e., extensive application to generated data with known characteristics or to actual behavioral data for which continuous recording has been carried out). In relation to the methodological abilities, it is crucial that students and applied researchers not only trust that the content taught by their teachers and textbooks is correct, but that they are aware that subject content is the result of research (e.g., via simulation) and that this research also presents certain limitations such as the ones mentioned in “Advantages and limitations of the application.” In summary, getting to know how knowledge is obtained is expected to make students and applied researchers exercise their critical thinking skills (although comprehensive programs are required for developing such skills; Halpern, 1998) and the disposition to always look for more refined and more precise knowledge.

### Limitations and Future Research

In terms of limitations, the present paper does not necessarily add new knowledge in terms of research findings. This is due to the fact that its purpose is mainly related to illustrating the complex relations of several factors influencing the accuracy of the estimates obtained via several observation recording procedures. Moreover, as previously mentioned, the factors included in the simulation do not include human error and one of its likely causes, fatigue. It could be logically argued that MTS entail smaller cognitive load (as attention is required only at the end of the interval), but fatigue is related to several additional factors such as the observer’s familiarity with the behavior, the interval length, the number of categories to be recorded, the average DPO of the behaviors and the degree to which they are easily distinguished (Altmann, 1974). Such information has to be considered, jointly with the evidence on the estimation of prevalence and frequency when selecting a RAUT.

Future illustrations can focus on study of reliability and, more specifically, agreement between observers. Rapp et al. (2011) showed how the values of percentage of agreement are different according to the observation recording procedure, but such illustrations are also necessary for kappa, which is recommended for quantifying agreement (Suen and Ary, 1989). Specifically, the kappa value obtained for continuous recording on a second-by-second comparison (Bakeman and Gottman, 1986) can be compared to the kappa values obtained via MTS, PIR, and WIR for varying degrees of prevalence of the behavior of interest, given that this parameter has impact on the kappa values (Suen and Ary, 1989).

## Author Contributions

The initial idea was due to JL and RM and it was further developed jointly by both authors. The manuscript was written by JL (Introduction) and RM (Method, Results, and Discussion). Both authors participated in several revisions during the process of creating, discussing, and improving the manuscript. Both authors gave their consent that this final version submitted for publication and agreed in their co-responsibility regarding all aspects of the work, such as the accuracy of the data and the integrity of the research.

## Conflict of Interest Statement

The authors declare that the research was conducted in the absence of any commercial or financial relationships that could be construed as a potential conflict of interest.

## Footnotes

1 Several possible distributions can be specified for the event durations and interim times, but we followed Pustejovsky and Runyon (2014) in using an exponential distribution.

2 www.shinyapps.io

3 http://cran.r-project.org

4 https://www.rstudio.com/products/rstudio/
